# The contribution of community health systems to resilience: Case study of the response to the 2015 earthquake in Nepal

**DOI:** 10.7189/jogh.13.04048

**Published:** 2023-05-12

**Authors:** Angeli Rawat, Asha Pun, KC Ashish, Indra K Tamang, Jonas Karlström, Katrina Hsu, Kumanan Rasanathan

**Affiliations:** 1 School of Population and Public Health, University of British Columbia, Vancouver, British Columbia, Canada; 2 UNICEF Nepal Country Office, Kathmandu, Nepal; 3 Department of Women’s and Children’s Health, Uppsala University, Sweden; 4 SingHealth Duke-NUS Global Health Institute, Singapore; 5 UNICEF, Health Section, New York, New York, USA; 6 Providence Health Care, Vancouver, British Columbia, Canada; **Background** Understanding how to build resilience in health systems is essential to ensuring countries can respond to shocks and has become increasingly important in the context of climate change. The 2015 earthquake in Nepal offered an opportunity to capture lessons learned and advance our understanding of resilience. Community members, especially female community health volunteers (FCHVs), were central to the response. We aimed to describe the successes and challenges with building resilience in community-based health systems after the earthquake response from multiple perspectives within the health system.

## Abstract

**Methods:**

Key informant interviews and focus group discussions were utilised. Participants included FCHVs, primary healthcare workers, community leaders and mothers, district health managers, representatives from the Ministry of Health and Population, multilateral health organisations, bilateral development partners, local non-governmental organisations, community-based organisations, and international non-governmental organisations. We used thematic content analysis to identify emerging themes.

**Results:**

Seventy-seven people participated in the study in September 2016 from communities (n = 53, 69%), districts (n = 8, 10%), and national levels (n = 16, 21%). Strong coordination, international and national support, and community engagement and participation were reported as successes of the earthquake response. Challenges included a lack of preparedness and supplies, a lack of earthquake-resistant infrastructure, and the centralisation of the response. FCHVs continued to work, despite being victims of the earthquake themselves. Facilitators of the continuation of the FCHVs’ duties included their strong ties with the communities and facilities, international support, and the ability to mobilise existing community resources. Barriers included fear, communities’ attitudes, high workloads, large geographic distances, and difficult geography. Participants identified the importance of having strong, connected, and supported communities, adaptable funding and policies, and decentralised decision-making within strong health systems.

**Conclusions:**

Building resilience in community-based health systems must start with strong communities that are prepared, trained, equipped, and empowered. Health systems must be decentralised and adaptable, with strong coordination and leadership. Capable community health workers such as FCHVs were an important part of building resilience during the earthquake. These lessons can assist countries in strengthening decentralised health systems to better respond to a multitude of shocks, while still providing essential health services for communities.

Understanding how to build resilience in health systems is increasingly urgent as countries with limited resources face multiple converging challenges, including threats from climate change and emerging pathogens. Global health threats such as the 2014 West African Ebola crisis and the COVID-19 pandemic have underscored the importance of building health systems’ ability to respond quickly and effectively to crises and mitigate their human, political, and economic impact [1-3]. Community-based health systems play a critical role in building resilience as the first point of contact with the health system; however, research on how to build their resilience in low- and middle-income countries (LMICs) is limited [4,5].

Despite the widespread use of the term “resilience”, its conceptualisation and operationalisation are still unclear [6-11]. Kruk et al. [12] define it as the “capacity of health actors, institutions, and populations to prepare for and effectively respond to crises; maintain core functions when a crisis hits; and, informed by lessons learned during the crisis, reorganise if conditions require it”. Some characterise resilience as an emergent property of health systems that uniquely captures the dynamic and interconnected nature of complex systems [11]. Others warn that the discourse on resilience idealises “bouncing back” while neglecting transformation and improvement, thus maintaining weaknesses that may have made the system vulnerable in the first place [13-15]. Placing the responsibility for adapting and responding to crises on individuals and communities has also been viewed as a form of neoliberal governmentality [16]. For the concept of health system resilience to be valuable, further research is needed to clarify how it can be defined and built into countries experiencing shocks.

The earthquake in Nepal offers an opportunity to address this gap. In April 2015, Nepal experienced a series of earthquakes and aftershocks, the largest of which registered a magnitude of 7.8. The earthquake’s epicentre was 181 km northwest of Kathmandu, in the Gorkha district [17]. The earthquakes and aftershocks affected almost half of the districts and resulted in nearly 9000 deaths, over 22 000 injured, and 2.8 million people displaced [18]. Over 1200 health facilities were destroyed or damaged, with some districts losing up to 90% of their health facilities [19,20].

Prior to the earthquake, Nepal’s health system faced chronic challenges, especially in the provision of maternal, newborn, and child health (MNCH) services [21]. Despite recent progress, Nepal still had the third highest maternal mortality ratio globally [22] and a high infant mortality rate of 32 per 1000 live births [23,24]. Inequitable access to services due to the country’s mountainous geography and limited transportation networks, and a lack of well-equipped health facilities with trained healthcare personnel further exacerbated existing inequities [23,25,26]. Lack of governance and financial management, low health system accountability to patients, and financial barriers for patients contributed to inequitable access to care [27]. During and after the earthquake, the health system was further pressured by increased rates of disability from injuries, ongoing mental health effects, and rising poverty, all occurring in the context of severe damage to health facilities [28].

Given these challenges, community-based health interventions such as those provided by female community health volunteers (FCHVs) in Nepal have become highly important. Since the 1980s, Nepal’s approximately 50 000 FCHVs have played an important role in improving MNCH [26,29]. FCHVs are typically women over the age of 25 who live within the communities and receive 18 days of basic training in family planning, MNCH, and nutrition [30]. They volunteer unpaid part-time, averaging 5-6 hours per week prior to the earthquake [26]. With over 80% of Nepal’s population living in rural areas [18], strengthening community-based interventions in partnership with FCHVs is key for improving healthcare access in hard-to-reach areas [23]. The role of the community health workforce is considered an integral, yet understudied, element of building resilience in health systems [31].

This study was part of a larger four-country study on understanding resilience in community-based health systems. We aimed to identify successes and challenges during the earthquake response to building resilience in community-based health systems. Sub-objectives included understanding barriers, facilitators, and changes to the roles of FCHVs, and identifying salient health system factors that could build resilience, especially at the community level.

## METHODS

We used key informant interviews (KIIs) and focus group discussions (FGDs) to meet our research objectives. KIIs and FGDs were semi-structured and facilitated by open-ended interview guides adapted to the participants’ level of knowledge of the health system based on three levels within countries (community, district, and national/international). We developed guides based on existing literature, as well as a conceptual framework that was modified based on local expertise from a health systems performance framework and included elements of the Tanahashi bottleneck analysis [32,33]. The resulting subthemes included successes and challenges of earthquake response on FCHVs, community engagement and participation, service delivery, sustainability, adaptability, preparedness, resources, governance and multisectoral collaboration, all from a health systems lens. We conducted FGDs at the community level with community members and health workers and KIIs with district, national, and international level participants.

We used purposive and snowball sampling techniques to identify and recruit participants with diverse perspectives from across the health system, including: FCHVs, mothers’ group members and members of the health facility management committees, primary healthcare unit staff, district health staff and managers, Ministries of Health managers, multilateral health organisations, bilateral development partners, local non-governmental organisations, community-based organisations, and international non-governmental organisations.

We approached participants in-person with a standard script explaining the study goals, the lead researchers’ institution affiliation, and the voluntary nature of participation in the language of their preference (e.g. English or Nepali). We collected written informed consent for all participants prior to participation. Participants who had low literacy provided thumbprints in lieu of signatures for written informed consent. For non-English speaking participants, a trained health worker performed simultaneous translation to allow researchers to further probe the participants and received their feedback. Translators had health backgrounds and were either UNICEF district staff or community health workers employed with implementing partners. No participants declined to participate in the study and there was no prior relationship between interviewers and study participants.

One researcher (AR) conducted the KIIs and FGDs in semi-private to private areas at health facilities, within communities, or at the offices of participants. KIIs lasted between 22 to 69 and FGDs between 26 to 86 minutes. All interviews were audio-recorded, transcribed verbatim, and translated into English (if in Nepali). Translators verified the accuracy of the simultaneous translation and noted potential discrepancies. For logistical reasons, we did not verify the transcripts with participants. There were no repeat interviews and no drop-out of study participants.

We analysed the data using a deductive-inductive approach. We conducted first-level coding of verbatim transcripts deductively based on the sub-objectives of the interview guides listed above. We then used thematic content analysis [33] based on the grounded theory approach [34] to identify emerging themes inductively. The grounded theory approach was chosen for its ability to identify the interconnectedness of the data and areas of conflict and contradiction. We utilised the ATLAS.ti software Version 7.5 (Berlin, Scientific Software Development) for data management and code organisation. We discussed any differences in interpretation until a consensus was reached among project team members. We then triangulated data across methods and by participant categories to understand relevant themes on building resilience in community-based health systems.

We received ethical approval from the University of British Columbia Behavioural Research Ethics Board (certificate H1502651) and the Nepal Health Research Council.

## RESULTS

Seventy-seven people participated in the study between 5 and 13 September 2016. Most participants (n = 53, 69%) were from the community level (i.e. mothers’ support groups, FCHVs, primary healthcare workers, health facility management committee) (**Table 1**). There were 53 participants in the FGDs and 24 in 17 KIIs.

**Table 1:** Description of participants from Nepal (n = 77)

	n (%)
**Community**	53 (100)
Female community health volunteers	9 (17)
Health facility operation and management committees	11 (21)
Mothers’ support groups and beneficiaries	23 (43)
Primary health care workers	10 (19)
**District**	8 (100)
District public health nurse	2 (25)
Supervisors/management	6 (75)
**National **	16 (100)
Ministry of Health representatives	4 (25)
UNICEF country office	7 (44)
Partners (bilateral, multilateral)	5 (31)
**Total**	77 (100)

### Facilitators to building resilience during the earthquake response

#### Active, dedicated female community health volunteers

The strong and adaptable roles of FCHVs were widely discussed as determinants of a successful earthquake response and in building resilience. District and community participants, especially FCHVs, described successes related to their evolving roles and associated training during the response (**Table 2**, Quote 1). Prior to the earthquake, FCHVs focused on MNCH (e.g. family planning, immunisations, antenatal care, promoting facility-based deliveries, nutritional screening for children, and postnatal care). After the earthquake, their roles expanded to include treating the injured, providing psychosocial support, empowering and mobilising communities, providing supplies, offering referrals to facilities, guiding medical teams, and monitoring and preventing outbreaks of infectious disease. Many participants, including FCHVs themselves, felt FCHVs’ intrinsic motivation to serve communities during the response and their high levels of job satisfaction were facilitators to their effectiveness (**Table 2**, Quotes 2,3). Participants described FCHVs as determined, motivated, and committed, and described how FCHVs continued to work despite being earthquake victims themselves – linking their involvement to reducing the impacts of the earthquake (**Table 2**, Quotes 4 and 5). Participants felt facilitators to their effectiveness were originating from the communities they served, which increased the respect communities had for them and their accountability to the communities, as well as being strongly connected with communities and the health system more broadly (**Table 2**, Quote 6).

**Table 2:** Successes of the earthquake response

Quote	Theme (perspective)	Quotation
1	Success of trainings (community – FCHVs)	*We got the opportunity to learn new things. We only knew some things superficially, but after the earthquake we got the opportunity to learn [them] in detail. We received training related to diseases among children. We learnt a lot and departed that knowledge in the village during meetings. We also had training related to mental health. After that, we have tried as much as we could, to send the sick to the health posts. –* FCHV
2	Motivated FCHVs (community – FCHVs)	*It’s been 25–26 years. We meet our friends here…like our name, we are volunteers after all. We got in the habit of serving the people. That became our religion. Because of that, we have been satisfied from within. –* FCHV
3	Motivated FCHVs (community – FCHVs)	*We have worked for so many years. We have been living together with the villagers and are happy with that. We feel that is easy. We work and for us money is not so important. Because we are working, we have been able to give nutrition to pregnant women and young children. We are happy and satisfied, because we have been saving lives. –* FCHV
4	Success of FCHVs being motivated despite being victims (national)	*After earthquake many Female Community Health Volunteers—their houses were badly destroyed. Despite that, they are not discouraged. They are encouraged. Actually what people observed were…. Without their house, they were involved to help people in treatments and for referrals. They were really involved and they were not discouraged. What we heard was that in spite of getting everything destroyed, they were teaching and taking care of patients. –* Ministry of Health manager
5	Success of FCHVs being motivated despite being victims (national)	*So they are themselves in the temporary shelter but they did a wonderful job so that we were not having heavy scale of outbreaks after the earthquake…we use them in the counseling part because they are the people who are nearby the households where the houses are damaged. There were no such systems of where to stay, how to make available daily foods, supplies, water. So they did that job wonderfully. –* Ministry of Health manager
6	Success of FCHVs in earthquake response (community)	*Our relationship with the FCHVs is excellent. If it was not for them, our programs would not be successful. It is only because they have been coordinating extremely well with us and the community people, that the programs have been successful. If they were not present, we could not have done it on our own. –* Health facility operation and management committee member
7	High community participation (national)	*What we learned [was] that our people are able to manage up to this level of the disaster and the community people showed their participation, engagement and management; not only in the health sector but also in providing the services to make a shelter available, water supply available, a toilet nearby the shelter. In those things community people are mainly involved. We have that participation—community thinking is very strong. –* Ministry of Health manager
8	Strong community participation (national)	*We all have experience that if [community management structures] are strong, the health facility become better and also they provide better service as well as much more accountable. –* Ministry of Health employee
9	District-level capacity to coordinate (district)	*The first lesson is the capacity of coordination. There should be strong coordination capacity of district health office. We have been successful due to strong coordination capacity, so, it must be developed. And the next thing is decision making authority to the district level the community level as well as resource also. –* District participant
10	International support building infrastructure (district)	*Donor supported organisations are investing in infrastructure because out of 52 health facilities, 46 were damaged, 33 were completely damaged, and 13 were partially damaged. They are supporting health facility construction and toilets, running water, electrification like solar system development and communication also mobile phones.* *–* District health officer
11	Trained districts in disaster risk reduction (international partner)	*And it was good that one of the district had just completed the disaster risk reduction process and they had already formed. They knew what they needed to do during disaster. So they also acknowledged because of this training, we were very fast to establish our committees in the district so we could work faster…but where it didn’t happen they didn’t know how to start, where to go. –* UNICEF country office

#### Support from community groups

Communities that were effectively engaged, proactive, and linked to their health systems were identified as central to building resilience and to a successful response. Many participants, especially community members, described how pre-established community groups engaged local leaders and gave communities a strong sense of ownership during the response. Many participants described how communities re-established health services and contributed their own resources during the response, often extending beyond the health sector (**Table 2**, Quote 7). Participants, including communities themselves, felt that good relationships between the communities and health facilities were large determinants of a successful response and that these relationships were supported by well-established health management committees and strong primary health posts (**Table 2**, Quote 8). Many participants described how agencies engaged healthcare workers (including FCHVs) to understand the needs of the community and felt that this led to strong community engagement and participation – qualities discussed as essential to building resilience. Participants also highlighted the role of the community groups to hold facilities accountable to meet the needs of the communities.

#### Strong district-level capacity and resources

The decentralisation of the response to the district level was discussed as building resilience by facilitating adaptability and timeliness during the earthquake response. Some national and district participants described the response as well-coordinated from the central level across districts and between districts, communities, and national levels, which led to less duplication of response efforts (**Table 2**, Quote 9). Timely support to District Health Officers (DHOs) by appointing senior officials from MoHP as a focal person for each district, making them accountable and dispatching these senior central level staff to the districts was also a determinant of the success. Participants felt quick information flow from the communities to national levels also helped with resource allocation.

#### International support

Many participants described international support (medicine, supplies, and health workers) from foreign medical teams and international non-governmental organisations as a success in the earthquake response. This support facilitated the treatment of complicated cases within communities by building infrastructure and supplies (e.g. hospitals, birthing centres, health facilities, instruments, and equipment) and re-establishing communication networks (**Table 2**, Quote 10). Training and engagement provided by organisations were also described as broadening FCHVs’ skill sets and validating their roles.

#### Preparedness

Preparedness and coordination were widely discussed needs for building resilience in health systems. Views varied on health system preparedness and communities’ success during the earthquake response. Many felt that the implementation of preparedness plans that were designed before the earthquake, both at the district level and within communities, was a large determinant of a successful response. Participants felt the districts where the government had pre-established district disaster management committees were quick in their response and were able to mobilise resources quickly and effectively (**Table 2**, Quote 11).

#### S*trong health systems and infrastructure*

One of the most widely discussed determinants of resilience within the earthquake response was a strong, well-resourced health system. Participants described how a strong primary healthcare system, free health services, transportation support, and strong referral linkages led to high immunisation rates and reduced maternal and neonatal mortality during the earthquake. Many participants felt the health system was stronger since the earthquake and felt that learning from it on how to build a stronger health system was key to building resilience.

### Barriers to building resilience

#### Increasing workloads among community health workers

Increasing workloads among community health workers after the earthquake, despite workers being victims themselves, was a widely discussed challenge to the response and building resilience (**Table 3**, Quotes 1 and 2) and was described as especially difficult given their voluntary role (**Table 3**, Quote 3). Inequitable distribution and overall lack of supplies and service availability within facilities placed challenges on FCHVs’ ability to perform their duties (**Table 3**, Quotes 4 and 5). Participants also highlighted the large distances that FCHVs had to travel across difficult terrain. Many participants recommended further empowerment, incentives, and training for FCHVs and expressed concerns about relying on a voluntary workforce to complete increasingly complex tasks with minimal training or education.

**Table 3:** Challenges to the earthquake response

Quote	Theme (perspective)	Quotation
1	Increased workload of FCHVs (FCHVs)	*The workload has increased significantly. After the earthquake, there have been a lot of programs. There is so much work added after the earthquake, like nutrition, mental health, and safe motherhood. Every program is linked with earthquake. –* FCHV
2	Increased workload of FCHVs from multiple engagements (district)	*They [FCHVs] have [an] overload of the job or responsibility after the earthquake because most of the programs the agencies first contact them for any programs – like health is the primary also women’s empowerment training, skill development training, or income generation programs. All depends upon the female community health volunteer, as a communicator, as a local facilitator –* District health officer
3	Voluntary cadre (community)	*Being volunteers, we have to inform people on every ward about a lot of things. Our work is difficult. We have to work at night too. If there are any pregnant women, we have to give them advice to take them to the hospital. All nine of us face the same problem. Every ward has the same difficulties. Because of that, we feel that a little incentive would give us some hope. –* FCHV
4	Inequitable supply distribution (community)	*But sometimes, there are sisters who live very far away… When NGOs [non-governmental organisations] and INGOs [international non-governmental organisations] need to distribute supplies for children and pregnant women, they call us to inform the villagers. But during those times, one problem is that not all the supplies are distributed and some people are left out. This burden falls on us when that happens. And considering the effort we put in our work, the incentives are not enough. –* FCHV
5	Lack of supplies/poor quality health services (community)	*All we do is give people counseling, and inform them that the services provided there [at the facility] are good, and that they will be safe there. But if there is no medicine or treatments there, how can it be safe? There is no point for us to keep advocating the services if there aren’t any. And we can’t provide such health services. –* FCHV
6	Need to support communities (national)	*Some kind of mechanisms should be there that the district region and national region can support them [communities] and help them to know their own strength. That is the starting step of resilience. –* Ministry of Health manager
7	Lack of district capacity (international partner)	*There is no capacity to deliver the medical interventions that are needed. District hospitals do not do even Caesareans. So when it came to injuries, close reduction of fractures, large lacerations which needed suturing and any kind of crush injuries, every patient had to be mobilised and brought to Kathmandu. –* Multilateral partner
8	District inflexibility of funding (international partner)	*Within our bureaucracy we don’t have the budget line item where there is flexible fund during emergency. For example, if there is emergency in place, can the district health office mobilise budget for the emergency? They can’t because they would be audited and if there is some kind of deviation from the routine activities then they would call it misappropriation or mis-utilisation of funds. So within the bureaucracy, we need a flexible line item for disaster management or preparedness –* UNICEF country office
9	One door entry for support at district level (district)	*What big lessons were…after earthquake, there was lots of goods, supplies supported from other agencies. But there was a one-door system. They have to come here in the DPHO [District Public Health Office]. Then they have to do entry here. Then after doing that, they can distribute. But now they are realising, that was their mistake. Because if they do distribution from different sites…different clusters…then it would be better. Then victim can easily rescued or we can support them very easily and very fast*. *–* District manager
10	International support – short expiry medications (national)	*Whatever the donations we received, what we realised is that whatever this list of commodities like drugs they supplied, that was a very short expiry and a huge quantity. Then that has to be disposed with the help of WHO. So if any international community, they want to donate anything, the list has to be forwarded to particular recipient then they could just select that this can be consumed, and this cannot be consumed –* Ministry of Health manager
11	Lack of preparedness in stockpiled commodities (international partner)	*The problem at the community level is that the medicine that supplies that were supposed to be there weren’t available so there was no stock piling of the equipment and commodities in place and that really hindered the response.* *–* UNICEF country office

FCHV – female community health volunteer, UNICEF – United Nations Children’s Fund

#### Lack of community-level leadership and engagement

Community members described how the fear of further aftershocks deterred community members from attending facilities and outreaches. District and national participants felt that there was a lack of strong leadership in communities and that village development committees may not be representative of the communities’ diversity. Community members felt there was a lack of autonomy for communities to use resources according to their priorities. Many participants highlighted the need for decentralised resources at the community level and improved community involvement in planning for earthquakes at the village, facility, and district levels. This included making the community aware of their roles and the resources available. Many participants also discussed the need for a mechanism for district, regional, and national levels of a health system to support communities on their own to build resilience (**Table 3**, Quote 6).

#### Inflexibility of a centralised response

Some national- and district-level respondents felt that the earthquake response was heavily dependent on capacity at the national level (Kathmandu) regarding supplies, commodities, hospitals, decision-making, and resource allocation (**Table 3**, Quote 7). This was seen as undermining the effectiveness of the district-level response. Participants also spoke of the emergency policy’s lack of flexible funding at the district level, which undermined districts’ ability to quickly mobilise resources and divert budgets during emergencies to increase adaptability (**Table 3**, Quote 8). Some participants discussed that the planning process was “bottom-up” in theory, but not in practice. Lastly, participants described the challenge of having all supplies go to the districts prior to dispatching into communities, an issue they felt delayed the distribution of supplies (**Table 3**, Quote 9).

#### Lack of coordination and effectiveness among international donors

The most widely discussed challenges were related to the changing landscape of donors after the earthquake, the limited capacity of the government to absorb donations, and top-down and politically-driven decision-making. Many participants discussed how the addition of many new actors during the response increased the complexity of planning health programs and led to duplication of services. District participants often described inappropriate district entry and that support from international partners was not aligned with the needs of the communities. Many participants felt that some donations were not useful to the local context and were either too technologically advanced (e.g. required technical expertise or further specialised equipment unavailable locally) or were in too large a supply to use in a timely way (e.g. large quantities of medicines close to expiry) (**Table 3**, Quote 10).

#### Lack of emergency preparedness

Many participants felt a lack of preparedness impacted the timeliness and effectiveness of the response. They discussed a lack of systems in place for food provision, supplies (e.g. medications), and shelter (e.g. replacement structures, identifying open spaces to relocate facilities). They also felt that stockpiled resources in warehouse systems and supply chains were inadequate, especially from districts to communities. This lack of preparedness was often cited as the cause of communities not knowing how to protect themselves and lacking medicine, equipment, supplies, or trained manpower (**Table 3**, Quote 11). Participants felt that setting up guidelines and protocols for the response took too long and should have been in place prior to the earthquake. Recommendations included investing in community structures and linking the health system to communities, more community-level planning, and training community members to know their responsibilities during a shock. Participants also suggested using drills across the health system and checklists at facilities to prepare the health system for earthquakes. The need for multi-year planning that is well-coordinated with all implementing bodies while considering integrating systems was also highlighted as necessary for building resilience.

#### Existing health system gaps

Many participants felt that weaknesses within the health system and beyond were exacerbated during the response. This included infrastructure (e.g. the lack of earthquake-resistant homes and facilities, mobile phone networks, electricity, roads, and water supply), equipment and supplies (e.g. lack of solar-powered refrigerators, medications, supply chains, and commodities for health service delivery), and human resources. National-level respondents felt the supply chain was now more fragmented and too focused on the injured at the cost of care to mothers and children. Many participants described the need for trained health workers, and more human resources with a mix of medical skills and non-skilled workers, especially primary healthcare managers.

## DISCUSSION

We identified multiple relevant themes on building resilience in community-based health systems from the experience of participants in Nepal during the earthquake response. Participants described several factors that contributed to or limited their communities’ capacity to respond to the earthquake and build resilience. Facilitators included active and dedicated volunteers who were linked to their communities, strong relationships between community groups and the health system, decentralised resources for a local response, well-coordinated international support, and proactive emergency preparedness. By contrast, barriers to the response included the increasing demands on unpaid volunteers, a lack of community-level leadership, inflexible centralised resource allocation, a lack of coordination among international donors, and a lack of proactive planning. Existing infrastructure gaps were also highlighted as an important part of emergency response; the areas that had stronger health systems in place were better able to respond, which becomes more crucial as health threats continue to emerge around the globe.

Community health workers, particularly FCHVs, played a central role in the success of the earthquake response in Nepal. FCHVs provided an important link between communities and their healthcare systems, but needed further training and support to be most effective during a shock, as discussed by others [34-36]. This is especially important for continuing the provision of and support for MNCH services, which are often impacted during shocks such as pandemics [37]. Specific considerations are needed to ensure a viable workload during shocks, and health worker safety must be prioritised, a finding reinforced by the experiences of healthcare workers in Nepal during the COVID-19 pandemic [38].

Participants also highlighted the importance of a decentralised crisis response. Communities should be empowered to be self-sufficient; decentralisation can enable this by encouraging the use of local initiatives, information, feedback, input, and control [39]. The importance of engaging community leadership in building resilience at the community level has been identified and prioritized in other post-earthquake contexts such as Haiti [40]. Ensuring pathways exist for communities to guide emergency response teams leads to a more timely and effective response, increases accountability to the needs of the communities, and ensures equity [39]. Our results indicate that communities need to be consulted as key partners in planning, prioritisation, and allocation of resources rather than merely during times of crisis. This is similar to what was found in Liberia in the post-Ebola virus disease context and has been described as increasing trust between communities and health systems [9,28,41]. Formalising relationships with key community groups and FCHVs, coupled with appropriate incentives, could relieve some of the burden posed by high workloads during shocks. However, to ensure the success of this community-led approach, political will must accompany decentralisation to ensure timely and effective responses.

Funding structures and policies must be adaptable and flexible in times of shock across multiple sectors and actors, which was also highlighted by Kruk et al. [11] as an indicator of a resilient health system. Participants in our study noted policies and funding structures did not allow for pre-positioned supplies and commodities or swift funding to communities and districts as their needs changed. Policies need to consider multiple actors in the health system – from international actors to districts and communities. This can prevent duplication of services and overburdening communities experiencing shock and coordinate international supply donations to where they are most needed. Creating policies that institutionalise the roles of FCHVs and community groups could facilitate a more coordinated response, as recommended elsewhere [30,42]. Although our study participants felt communities and the healthcare system were more prepared for a shock following the earthquake, the COVID-19 response in Nepal was hindered by poor coordination and governance, highlighting the ongoing need for increased flexibility and preparedness for multiple threats [43].

Health systems can only be resilient in a time of shock if they are strong, prepared, equitable, and well-resourced. This includes ensuring human resources are sufficient in number, geographic distribution and skills mix within both facilities and communities to be effective during shocks and thereafter. A recent analysis of Nepal’s primary healthcare system found the system continues to be constrained by a shortage of skilled human resources, the impacts of which will be exacerbated during times of shock [28]. In addition, interactions with complex systems outside the health system must be considered, as discussed by Barasa et al. [44]. Adequate electricity, earthquake-resistant buildings, safe and accessible water and roads, strong communication networks, and strong supply chains must be considered when building resilient health systems to ensure continued service delivery during shocks.

This study has several strengths and limitations. One of its strengths is the diversity of participants from across the health system, including a variety of participants at the community level, allowing us to bring the experiences of those on the ground into the discussion on building healthcare system resilience in community-based health systems. Nepal’s health system is unique where the FCHV program has been providing care to communities for over three decades. This provides unique evidence and highlights challenges that persist compared to more nascent community health worker programs. Some limitations of the research exist as well. Our data was captured in September 2016 (17 months after the earthquake) and therefore may not represent the successes and challenges immediately following the earthquake in April 2015 and may be susceptible to recall bias. Additionally, we may have captured a unique time in the recovery process that may not be generalisable to the post-earthquake period, as resources and international support may have waned. Further research is needed to understand how healthcare systems and communities at large build resilience at multiple stages after a shock and how many of those changes lead to stronger healthcare systems in the long term.

## CONCLUSIONS

Building resilience in community-based health systems must start with strong communities that are prepared, trained, equipped, and empowered. Healthcare systems must be decentralised and adaptable and have strong coordination, preparedness, supportive policies and leadership. Capable community health workforce such as FCHVs were a large determinant of building resilience during the earthquake and should continue to be supported. As climate change accelerates the frequency of natural disasters and related health challenges in Nepal [45], building resilience at the community level is essential for future ability to withstand and recover from shocks.
